# Type I Interferons as Regulators of Lung Inflammation

**DOI:** 10.3389/fimmu.2017.00259

**Published:** 2017-03-10

**Authors:** Spyridon Makris, Michelle Paulsen, Cecilia Johansson

**Affiliations:** ^1^Section of Respiratory Infections, National Heart and Lung Institute, Imperial College London, London, UK

**Keywords:** lung, infection, inflammation, type I interferons, pattern recognition receptors

## Abstract

Immune responses to lung infections must be tightly regulated in order to permit pathogen eradication while maintaining organ function. Exuberant or dysregulated inflammation can impair gas exchange and underlies many instances of lung disease. An important driver of inflammation in the lung is the interferon (IFN) response. Type I IFNs are antiviral cytokines that induce a large range of proteins that impair viral replication in infected cells. This cell-intrinsic action plays a crucial role in protecting the lungs from spread of respiratory viruses. However, type I IFNs have also recently been found to be central to the initiation of lung inflammatory responses, by inducing recruitment and activation of immune cells. This helps control virus burden but can cause detrimental immunopathology and contribute to disease severity. Furthermore, there is now increasing evidence that type I IFNs are not only induced after viral infections but also after infection with bacteria and fungi. The pro-inflammatory function of type I IFNs in the lung opens up the possibility of immune modulation directed against this antiviral cytokine family. In this review, the initiation and signaling of type I IFNs as well as their role in driving and maintaining lung inflammation will be discussed.

## Lung Inflammation

Mucosal surfaces such as those found in the intestine and the lungs are the most common targets for invading pathogens. The surface area of the human lung is approximately 70 m^2^, and its main function is gas exchange (1). The lung is constantly in direct contact with the environment and the cells of the lung need to be able to tolerate non-harmful stimuli but react appropriately to harmful pathogens. When lung cells respond to invading pathogens, the regulation of inflammation is particularly important since the lung comprises delicate structure crucial for conducting gas exchange.

Interferons (IFNs), discovered in the 1950s, represent a family of cytokines, which induce robust antiviral and immunomodulatory responses to interfere with virus replication and spread (2–4). IFNs can be classified into three main subclasses: type I, II, and III. Type I IFNs consist of several IFN-α isoforms (13 in human and 14 in mice), IFN-β, IFN-ɛ, IFN-κ, and IFN-ω. In contrast, type II IFNs include only one member, IFN-γ. The most recently discovered group of IFNs are the type III IFNs, including IFN-λ1 (IL-29; non-functional pseudogene in mice), IFN-λ2 (IL-28A), IFN-λ3 (IL-28B), and the very recently described human IFN-λ4 (5–7). This review will focus on the role of type I IFNs in the lung, however, many of the effects induced by type I IFNs will also be mediated by the other types of IFNs.

Sensing of pathogen-associated molecular patterns (PAMPs) by pattern recognition receptors (PRRs) results in production of many cytokines and chemokines including the type I IFNs. These cytokines have a whole array of functions. First, they elicit an antiviral state in infected and neighboring, uninfected cells. Second, type I IFNs modulate the immune response by promoting antigen presentation, cytokine production, dendritic cell (DC) and natural killer (NK) cell activity, and macrophage function. Third, they enhance the adaptive immune response by manipulating T cell effector function and antibody production. Overall, the antiviral and immune stimulatory potential of type I IFNs is required for the effective clearance of acute viral infections (2–4, 8). Furthermore, the impact of type I IFNs on the inflammatory response during other types of infections is also starting to be appreciated.

## Induction of Type I IFNs

**Figure 1:**
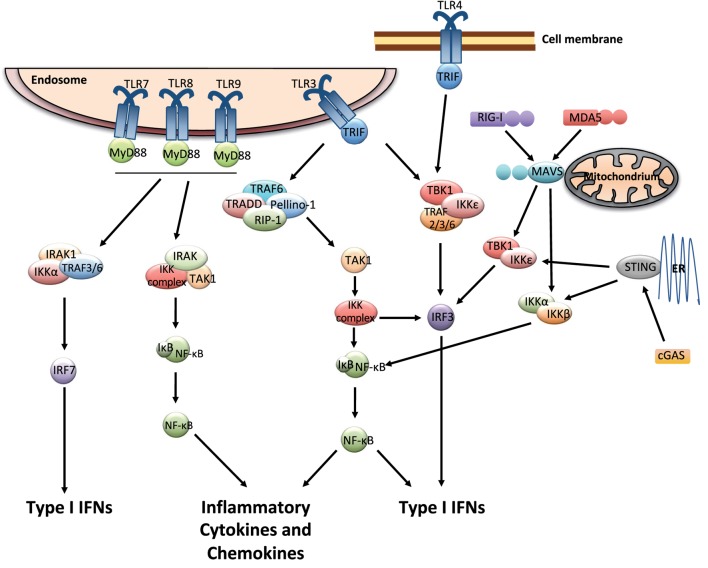
**Pattern recognition receptor signaling that leads to the induction of type I interferons (IFNs)**. The endosomally expressed TLR-3, -7, -8, and -9, cell surface expressed TLR4, the RLRs [retinoic acid-inducible gene I (RIG)-I and MDA-5], and cGAMP synthase (cGAS) can couple pathogen detection to type I IFN induction. TLR3 and TLR4 signal *via* TRIF, which occurs through inhibitor of kappa-B (IκB) kinases (IKKs), tumor necrosis factor (TNF) receptor-associated factor (TRAF) family associated NF-κB activator (TANK)-binding kinase-1 (TBK1), and IKK-ɛ. This causes the activation of IRF3, which in turn induces the expression of type I IFNs. The activation of TLR3 can also induce the production of inflammatory mediators *via* TRIF by activating a complex formed by TRAF-6, TNF receptor type I DEATH domain-associated protein (TRADD), Pellino-1, and the receptor-interacting kinase (RIP)-1. This causes the activation of NF-κB pathway, which is mediated by the IKK complex and transforming growth factor beta activated kinase (TAK)-1. TLR7, 8, and 9 use MyD88 for downstream signaling and can activate IRF and NF-κB pathways. RIG-I and MDA5 signal through the adaptor molecule mitochondrial antiviral signaling protein (MAVS). cGAS signals *via* the adaptor protein stimulator of interferon genes (STING). MAVS and STING further recruit signaling molecules (involving the IKK complex, TBK1, and several TRAF proteins) and lead to the activation of NF-κB and IRF3, resulting in gene expression of various antiviral cytokines including type I IFNs (9–13).

Multiple cell types including leukocytes and structural cells can detect PAMPs, in various cellular compartments and in all tissues. Recognition of PAMPs by the PRRs initiates an intracellular signaling cascade that causes the translocation of transcription factors to the nucleus initiating innate immune gene expression. The PRRs that can couple pathogen detection to type I IFN induction are toll-like receptors (TLRs), retinoic acid-inducible gene I (RIG)-I-like receptors (RLRs), and cGAS/cGAMP/stimulator of IFN genes [stimulator of interferon genes (STING); Figure 1].

The endosomally expressed TLRs that induce type I IFN gene expression are TLR-3, -7, -8, and -9, each of which detects different forms of nucleic acids (14). TLR3 recognizes double-stranded (ds) RNA which initiates the TRIF-dependent signaling cascade (15). TLR-7/8 and -9 recognize GU-rich single-stranded (ss) RNA and unmethylated CpG DNA, respectively (9), and they require MyD88 for their signaling. Finally, one cell surface expressed TLR, TLR4, which recognize lipopolysaccharides (LPS), and respiratory syncytial virus (RSV) F protein (16), can induce type I IFNs *via* TRIF-mediated signaling (15) (Figure 1).

The three receptors in the RLRs family, RIG-I, melanoma differentiation-associated protein 5 (MDA5), and laboratory of genetics and physiology 2 (LGP2) detect viral RNA in the cytosol (10, 11). Due to their localization within the cytoplasm, these receptors are constantly exposed to host RNA, and it is therefore important to distinguish it from that of viral origin. For this reason, the receptors can detect features that are common in many viral genomes and viral replication intermediates but absent from the host (12). RIG-I specifically binds to short uncapped 5′-triphosphate (5′-ppp) and 5′-diphosphate (5′-pp)-bearing base-paired RNA molecules, an RNA motif known to be present in some viral genomes but not in host RNA (17–19). MDA5 binds long stretches of base-paired RNA, which, again, are absent from uninfected cells but often produced as a consequence of viral replication (11). Structurally, RIG-I and MDA5 share many similarities as they both have caspases activation and recruitment domains (CARDs) essential for the downstream signaling (10, 11). LGP2 possesses the RNA-binding domain but it lacks the CARD domains and is therefore not involved in direct signaling. Instead, a role for LGP2 in assisting MDA5-mediated signaling has been suggested (20). For both RIG-I and MDA5, binding of agonists allows downstream signaling through the adaptor molecule mitochondrial antiviral signaling protein (MAVS), leading to the activation of NF-κB and IRF3 and subsequent induction of gene expression of various antiviral mediators including type I IFNs (11, 21, 22) (Figure 1).

The first DNA sensor described was DNA-dependent activator of IFN-regulatory factors (DAI), which was shown to induce type I IFNs during *in vitro* infections of fibroblast with either herpes simplex virus 1 or cytomegalovirus (CMV). However, the *in vivo* role for DAI remains unclear (23). Another pathway of recognizing infections with DNA viruses is *via* RNA polymerase III (PolIII), which transforms DNA into a 5′-ppp RNA, the ligand for RIG-I, yet the physiological role of PolIII remains elusive (24, 25). Other proteins belonging to the PYHIN (IFI16; IFN gamma-inducible protein 16, and AIM2; absent in melanoma 2) or DExD/H-box helicase (DDX) protein families were suggested likely DNA sensors, but their specific role *in vivo* is unclear and is being investigated [(26); for an extensive review about DNA sensing, see Ref. (24)]. The discovery of the adaptor protein STING identified a pathway crucial for the recognition of foreign dsDNA *in vivo*. Located at the endoplasmic reticulum, STING activates signaling *via* NF-κB and IRF3, resulting in the production of type I IFNs (27) (Figure 1). Recently, guanosine-monophosphate adenosine-monophosphate (cyclic GMP-AMP or cGAMP) synthase (cGAS) was identified as a DNA sensor leading to the downstream activation of STING. cGAS binds dsDNA and catalyzes the synthesis of the second messenger cGAMP from ATP and GTP, which binds to STING and activates the signaling cascade leading to type I IFN production (28–30). Interestingly, cGAMP can be transferred through gap junctions from infected cell to neighboring cells and thereby spread antiviral immunity (31).

Different cells of the lung will respond differently to infections depending on both the tropism of the pathogen (which cells that are infected) and which PRRs that are triggered. Lung epithelial cells are the first and most abundant cell type that will interact with the pathogens, and they have the ability to induce IFN-β production especially after influenza virus infection (32). In addition, plasmacytoid dendritic cells (pDCs) have a constitutive expression of IRF-7, which make them able to respond rapidly to TLR7 ligation and induce type I IFNs (33). This is especially important during influenza virus infection (34). However, during RSV infection, it is the alveolar macrophages (AMs) that are the major source of type I IFNs and they use MAVS-dependent PRRs for sensing the virus (35). Thus, in the lung as well as on other sites, many different pathogens can be recognized by several sets of PRRs expressed on the cell surface, in endosomes, or in the cytosol by different cell types. In combination, this will result in the induction of type I IFNs and an efficient antiviral response.

## Signaling through the Type I IFN Receptor

**Figure 2:**
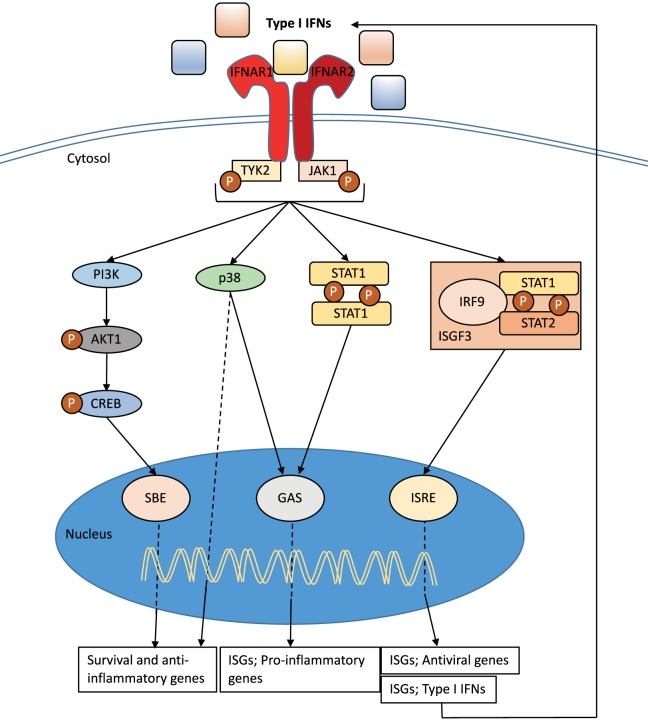
**Type I interferon (IFN) signaling**. Type I IFNs bind to the heterodimeric transmembrane IFN-α/β receptor (IFNAR), which is composed of the two subunits: IFNAR1 and IFNAR2. The c-termini of IFNAR1 and IFNAR2 are associated with the tyrosine kinase 2 (TYK2) and Janus kinase 1 (JAK1), respectively, and activation of the receptor transduces the phosphorylation of JAK1 and TYK2 by tyrosine phosphorylation. This initiates a signaling cascade composed of proteins of the signal transducer and activator of transcription (STAT) family. The STAT1 and STAT2 proteins are activated upon JAK1 phosphorylation, dimerize and together with IRF9, form the ISG factor 3 (ISGF3) complex. This complex translocates to the nucleus and binds to IFN-stimulated response elements (ISREs) in interferon-stimulated genes (ISGs) promoters to initiate gene transcription. Signaling through IFNAR can also occur independent of IRF9 recruitment through STAT1 homodimers that can bind to IFN-γ-activated sites (GAS) in ISG promoters. Both pathways initiate transcription that promotes the induction of a range of pro-inflammatory mediators and enhance the antiviral state. The JAK–TYK signaling pathway can also promote signaling pathways independent of STAT signaling. One such pathway includes MAPKs, which are important for signals regulating important cellular functions such as gene transcription, post-transcription, apoptosis, and cell-cycle progression. Specifically, the p38 signaling cascade after IFN-stimulation drives transcription of genes that are important for inducing the antiviral effects of type I IFNs and are regulated by ISREs and GAS. Further to MAPK, the type I IFN receptor signaling can also activate the phosphoinositide 3-kinase (PI3K) signaling pathway. The phosphorylation of PI3K causes the activation of the RAC-α serine/threonine-protein kinase (AKT1)/cAMP responsive-element-binding protein that can bind smad binding elements (SBE). This signaling pathway is believed to be important for transcription of genes controlling cellular survival and inflammatory (36, 38–40).

Type I IFNs bind to the heterodimeric transmembrane IFN-α/β receptor (IFNAR), which is expressed on all nucleated cells and composed of the two subunits: IFNAR1 and IFNAR2 (36) (Figure 2). *Via* signaling through the Janus kinase (JAK)/signal transducer and activator of transcription (STAT) pathway, the induction of several hundreds of interferon stimulated genes (ISGs) is initiated (36–40) (Figure 2). These ISGs interfere with various stages of the viral cycle and change the state of the infected and neighboring cells. Type I IFN signaling also drive the immune response to a number of pathogens by, for example, enhancing the production of inflammatory mediators, cell communication, and the induction of apoptosis in infected cells (see below). It is interesting that the type I IFN receptor has so many ligands, and it is still unclear if all type I IFNs bind the receptor in the same way or if binding of certain IFNs results in a functional difference. There is evidence that different IFNs bind to different anchor points resulting in variations to the binding affinities and conformational change of the IFNAR1 (41). In addition, IFN-β can ligate only the subunit IFNAR1 and signal independently of JAK–STAT pathways (42). Type I IFN production and signaling are tightly regulated by a positive feedback loop, with early IFNs (in the mouse IFN-β and IFN-α4) stimulating the expression of the ISG IRF7 and other important signaling molecules (43). This regulates the expression of all IFN-α isotypes (44) resulting in enhanced signaling through the type I IFN receptor (Figure 2). While the positive feedback loop is important for enhancing the production and effect of type I IFNs, equally important are the negative regulators that are required to restore cellular homeostasis. The type I IFN response is tightly controlled by a series of mechanism that are dependent on cell-intrinsic factors, ISG-mediated proteins, and miRNA. The activity of STAT proteins can be regulated by protein inhibitors of activated STAT (PIAS) and the suppressor of cytokine proteins (SOCS). Another important ISG for the regulation of type I IFN signaling is the ubiquitin carbol-terminal hydrolase protein, USP18 (40, 45).

Type I IFN responses are difficult to study in humans since by the time patients with severe lower respiratory tract infections are likely to be admitted to hospital several days have passed since the initial infection, at which time the production of type I IFNs is declining and not easily detectable. However, it is interesting that complete type I IFN deficiency has not been described in humans but mutations in STAT1, tyrosine kinase 2, or NEMO are associated with poor control of viral infections (46, 47). Recently, a mutation in IRF7, leading to impaired type I and type III IFN responses, was shown to result in severe influenza infection in one case (48). In addition, severe RSV disease has been associated with polymorphisms in several innate immune response genes, in particular many that control the type I IFN system (49, 50). Also, several ISGs have been associated with severe influenza infection in mouse and man, for example, IFITM3 and MX1 (51–54). Thus, most data suggest an important role for type I IFNs during respiratory infections.

## Type I IFNs Driving Lung Inflammation

The type I IFN signaling is especially important for the control of viral infections. However, in many diseases, the usefulness of type I IFNs has been debated (55). Like all immune responses, a balance is required and launching a response is as important as dampening it. As type I IFNs can lead to both cellular recruitment and activation, an imbalance of the type I IFN response can influence the cellular responses to either result in immunosuppression or immunopathology.

Type I IFNs have multiple effects in the lung. They have been shown to result in the production of chemokines such as CCL2, a monocyte chemoattractant, and CXCL10, important for the recruitment of monocytes/macrophages, T cells, NK cells, and DCs, therefore directly influencing inflammation in the lung (35, 56–58). The type I IFNs also drive a multitude of events in DCs including the differentiation of precursors, increased antigen presentation and cross-presentation, expression of costimulatory molecules, and promoting chemokine secretion and migration (8, 59–66). Interestingly, different subsets of DCs have a different degree of sensitivity to type I IFN signaling, which determines the susceptibility to influenza virus infection and thereby how much antigen they can present (67). Type I IFNs are also important for the activation of macrophages and NK cells (8, 68). Also, CD4^+^ and CD8^+^ T cells are directly affected by type I IFNs during various infections and the effect of type I IFNs can either be stimulatory or inhibitory by stimulating proliferation, differentiation, survival as well as inducing anti-proliferative and pro-apoptotic responses (63, 69). Type I IFN receptor signaling in T cells is also important for cytokine secretion, cytotoxicity, and memory formation (63). This varied effect of type I IFNs on T cells is partly dependent on the different STATs induced by type I IFNs where for example STAT1 is pro-inflammatory, pro-apoptotic, and anti-proliferative, while STAT4 promotes proliferation and cell survival (63). The different effects are dependent on when and where the T cells get the signal *via* the type I IFN receptor and will therefore be dependent on the site of infection, timing, magnitude, and source of type I IFN responses. B cells are also influenced by type I IFNs, and type I IFN receptor signaling on both B and T cells is important for antibody production (70, 71). Interestingly, it has been suggested that during LCMV infection, type I IFN receptor signaling in CD8^+^ T cells increase their killing of B cells and therefore decrease the production of neutralizing antibodies (72). If this is also occurring during respiratory infections remain to be elucidated.

In the absence of type I IFNs, there is often less inflammation in the lung. Interestingly, intranasal administration of IFN-α alone can stimulate the expression of pro-inflammatory cytokines (56, 73). Also, IFN-α can potentiate the pro-inflammatory responses after intranasal LPS challenge (56). How this occurs considering most cytokines and chemokines are induced by NF-κβ and not IFNAR signaling is unknown. One possibility is the fact that type I IFNs increase the expression of many PRRs or related signaling molecules, which can enhance signaling *via* other pathways for example those involved in NF-κB activation (74). Nevertheless, all cellular components involved in the mutual influence of NF-κB and type I IFN pathways are not fully investigated and might depend on the cellular context. It is therefore a likely scenario that type I IFNs play a role in NF-κB-induced expression of pro-inflammatory cytokines and chemokines.

### Lung Viral Infections

Type I IFNs are rapidly produced during all lung viral infections and contribute to the initial control of viral replication before effective innate and adaptive cellular responses are generated to clear the virus. Signaling *via* the IFNAR activates a cascade of ISGs that directly interfere with viral replication and viral spread. These include proteins that inhibit virus entry (e.g., MX1, IFITM proteins, TRIM proteins), modulate membrane lipids to prevent viral release (e.g., Viperin, Tetherin), induce apoptosis of infected cells [e.g., protein kinase R (PKR)], regulate transcriptional (e.g., Viperin) and posttranscriptional (e.g., OAS/RNaseL, PKR) mechanisms, and posttranslational events (e.g., ISG15) (38, 40). In addition to the ISG-mediated effects, type I IFNs modulate cell viability and function (e.g., apoptosis, inhibiting cell death, differentiation, migration, proliferation) to support antiviral defense (75). The amplification of the primary signal by type I IFNs is achieved by upregulation of many PRR molecules and associated signaling molecules like TLR3, RIG-I, MDA5, MAVS, MyD88, IRF3, and IRF7, which are themselves ISGs and therefore amplify the type I IFN response to viral infections [see above and Ref. (76, 77)]. Without a functioning IFNAR loop the detection of accumulating viral RNA and the further downstream processing of the signal is compromised in infected cells as they lack the feedback-mediated boosted expression of the viral RNA sensors. This imbalance will eventually promote viral replication and spread early during infection and also influence the degree of inflammation. For example, in a severe acute respiratory syndrome-CoV infection, type I IFN induction is delayed resulting in an overwhelming viral burden (78). Interestingly, many viruses have virulence factors that antagonize type I IFN responses [reviewed elsewhere (79–81)], indicating the importance of type I IFN responses during viral infection. It is also important to note that, especially during influenza virus infection, type III IFNs (IFN-λ) are highly induced at the same time as type I IFNs (82, 83). Interestingly, the receptor for IFN-λ is mostly expressed by epithelial cells and therefore these cytokines have a more restricted effect directed to intrinsic antiviral mechanisms (73). The effects of IFN-λ have recently been reviewed (5).

There are also age-related effects of type I IFN production in response to respiratory infections as both cells from infants and neonatal mice show a reduced type I IFN production and ISG induction after RSV exposure (84–86). Furthermore, monocytes from elderly have a diminished type I IFN response after exposure to influenza virus (53). Interestingly, these age groups are also very vulnerable to respiratory infections.

In addition to the cell-intrinsic responses, type I IFNs are known to enhance immune responses especially by activating and recruiting immune cells. During RSV infection, type I IFNs produced by AMs, induced the production of CCL2 and other chemoattractants crucial for monocyte extravasation into the lung during RSV infection (35). These recruited monocytes are important for controlling the virus (35). Interestingly, the expression of pro-inflammatory cytokines and chemokines was significantly reduced in RSV, influenza virus, and human metapneumovirus-infected *Ifnar1^−/−^* mice (56, 87, 88). This highlights again a synergizing effect of NF-κB and type I IFN pathways in inducing the optimal secretion of cytokines and chemokines. However, NF-κB translocation into the nucleus can be detected as early as 0.5 h post RSV inoculation without the necessity of viral replication (89). This might provide an explanation for the early induction of some of the measured cytokines such as IFN-β, IL-6, TNF-α, or IL-12 in the lungs of RSV-infected *Ifnar1^−/−^* mice, but perhaps sustained NF-κB activation may be in part type I IFN dependent. In contrast to *Ifnar1^−/−^* mice, wt mice have the ability to further enhance this “first signal” through the IFNAR-driven autocrine and paracrine amplification loop to maximize the responses. Moreover, the lack of responsiveness to viruses in *Ifnar1^−/−^* mice could also suppress the recruitment of additional cells to the site of infection, which could otherwise contribute to local cytokine secretion. These usually specifically recruited cells are most likely of non-polymorphonuclear origin, since neutrophil recruitment was comparable or increased in RSV or influenza virus infected *Ifnar1^−/−^* mice compared to wt mice (56, 90). Furthermore, during influenza or Sendai virus infection type I IFNs can act as messengers from the lung to the bone marrow where they instruct leukocytes to activate an antiviral transcriptional program, resulting in an increased antiviral state of these cells before they migrate to the lung (91).

Additional to T cell expansion, type I IFNs have been shown to promote T effector cell function due to the dependence on IFNAR mediated STAT1 signaling for the cytolytic activity of memory CD8^+^ T cells during recall responses with respiratory viruses (92). In contrast, after influenza virus infection type I IFNs can induce the secretion of IL-10 from CD8^+^ T cells (93) and the expression of programmed cell death ligand 1 (PDL-1) on epithelial cells (94). Furthermore, blocking of PDL-1 enhanced CD8^+^ T cells function and viral clearance (94). Thus, type I IFNs are important both to induce T cell effector functions and also to induce anti-inflammatory mechanisms that can suppress the T cell response.

Despite the essential role for type I IFNs in activating the immune response to successfully combat viral infection and to guarantee survival of the organism, they can have deleterious effects on the host and cause acute immunopathology. High type I IFN production during influenza virus infection mediated by recruited inflammatory monocytes and pDCs cause the upregulation of TNF-related apoptosis-inducing ligand (TRAIL) (8, 95). This “death” ligand binds to the death receptor 5 on epithelial cells, the expression of which is also upregulated by type I IFNs. Thus, the TRAIL-expressing inflammatory monocytes cause the death of the epithelial cells, which in turn increases morbidity or death of the mice (83, 96). Therefore, the potential of type I IFNs to drive and amplify pro-inflammatory responses (56, 83) could, if type I IFNs are produced excessively or for an extended time, cause the increased morbidity and mortality during lung viral infections (78, 83, 96). Thus, the timing and magnitude of type I IFN responses are crucial to obtain an efficient cell-intrinsic response and a balanced cell-extrinsic response that together lead to the clearance of the virus without causing detrimental immunopathology.

### Lung Bacterial Infections

Since type I IFNs have an important role in preventing replication and spread during viral infections their role is overall beneficial. During lung bacterial infections, type I IFNs are induced but the role of these cytokines is unclear and their ability to drive inflammation might in these cases be more detrimental. For example, mice deficient in IFNAR1 or TLR9 showed an improved clearance of *Staphylococcus aureus* (97). Also, a lung infection model of *Chlamydia trachomatis* mouse pneumonitis (*Chlamydia muridarum*) showed that deficiency in the IFNAR1 resulted in less bacterial burden and bodyweight loss, and milder pathological changes (98). *Mycobacterium tuberculosis* (MTb) infection of TB-susceptible *Ifnar1^−/−^* mice showed enhanced protection from death, lower bacterial burden in the lungs, and decreased degree of lung inflammation compared to wt mice (99). In addition, an interferon signature is evident in patients with active MTb disease (100), and mouse studies have shown that the type I IFN responses during MTb infection is tightly regulated by IL-1 and PGE_2_ (101). More contradictory data are presented for *Streptococcus pneumoniae* infection, where an invasive strain of *S. pneumoniae* induces type I IFNs in the lungs and blocking the IFNAR decreased the systemic bacteremia (102). In contrast, another study showed that *S. pneumoniae* infection of *Ifnar1^−/−^* mice or mice treated with an antibody against the type I IFN receptor displayed enhanced bacterial spread and increase bacteremia (103). In addition, if mice were given rIFN-β, this reduced the bacteremia after intranasal *S. pneumoniae* infection (103). Thus, most data suggest that type I IFNs induced during lung bacterial infection are part of the inflammatory response and might be important to initiate immune responses to the infection. However, since these cytokines lead to the recruitment and activation of immune cells, this can enhance inflammation and result in bacterial dissemination and spread.

Interestingly, bacterial secondary infections are common after a severe lung viral infection and the host is more susceptible to infection by *Escherichia coli, Pseudomonas aeruginosa, S. pneumoniae*, or *S. aureus* after influenza virus infection (104–106). This increased susceptibility is probably due to many factors such as a lower activation threshold of lung cells, inhibition of important signaling pathways and cytokines induction and exhaustion of immune cells resulting in non-appropriate immune responses elicited to the new pathogen. Noteworthy, this increased susceptibility cease to exist as soon as the virus-induced type I IFNs are decreasing (104) and IFNAR1 deficient mice can effectively clear a secondary *S. pneumoniae* infection (106). Furthermore, the induction of type I IFNs during influenza virus infection attenuates chemokines important for neutrophil recruitment, which can promote secondary bacterial pneumonia (107). A possible mechanism for this is the type I IFN-dependent upregulation of the methyltransferase Setdb2, which can repress chemokines such as CXCL1 at the chromatin level (108). Also, influenza virus infection before or during MTb infection increases the severity of the MTb infection *via* type I IFN signaling (109). Interestingly, type I IFNs have been shown to inhibit inflammasome activation and IL-1 responses and increase IL-10 production (110). Altogether, this suggests that type I elicited during a lung viral infection makes it more possible for a subsequent bacterial infection to establish.

### Lung Fungal Infections

The role of type I IFNs during fungal infections has also been investigated using mice deficient in the IFNAR1. During a lung *Pneumocystis* infection, a decreased pro-inflammatory response was detected and even if the pathogen burden was the same, the clearance was delayed in *Ifnar1^−/−^* mice and this resulted in an exacerbated Th2 response and fibrosis (111). The lack of type I IFN signaling during *Cryptococcus neoformans* infection has been shown both to result in a decreased pathogen burden (112) and increased pathogen burden with increased death (113). However, both studies showed higher Th2 cytokine levels in the *Ifnar1^−/−^* mice after *C. neoformans* infection (112, 113). Thus, type I IFN responses during lung fungal infection are also part of driving the inflammatory response but it is still unclear in which magnitude or which mechanisms that are used.

## Summary

There are obvious benefits of type I IFNs during a lung viral infection as these cytokines have a vital cell-intrinsic antiviral effect limiting viral replication. However, the cell-extrinsic effects of type I IFNs are important during all lung infections as type I IFNs directly drive lung inflammation, most likely by amplifying primary signals initiated by other stimuli. When considering to use type I IFN as a potential antiviral agent, the immune-modulating effects during lung infections needs to be considered as the type I IFN response has to be tightly regulated so that a balance of beneficial (initiation of inflammation) and detrimental (immunopathology) effects is achieved and gas exchange is not impaired.

## Author Contributions

CJ conceptualized the scope of the review article. CJ, SM, and MP wrote the review.

## Conflict of Interest Statement

The authors declare that the research was conducted in the absence of any commercial or financial relationships that could be construed as a potential conflict of interest.
